# [Corrigendum] Ursolic acid protects against cisplatin-induced ototoxicity by inhibiting oxidative stress and TRPV1-mediated Ca^2+^-signaling

**DOI:** 10.3892/ijmm.2026.5789

**Published:** 2026-03-09

**Authors:** Yang Di, Tao Xu, Yuan Tian, Tingting Ma, Donghao Qu, Yan Wang, Yuhan Lin, Dongyan Bao, Li Yu, Shuangyue Liu, Aimei Wang

Int J Mol Med 46: 806-816, 2020; DOI: 10.3892/ijmm.2020.4633

Following the publication of this paper, it was drawn to the Editor's attention by an interested reader that, regarding the immunohistochemical images in Fig. 2A on p 809, the Control/SG and UA/SG data panels contained an overlapping data section, suggesting that these data panels had been derived from the same original source. In addition, concerning the outer hair cell images shown in Fig. 4A on p. 811, the CDDP/TRITC and UA+CDDP/TRITC data panels appeared to be matching, suggesting that this figure had also been assembled incorrectly.

Upon contacting the authors about these issues, they realized that Figs. 2 and 4 in this paper had inadvertently been assembled incorrectly. The revised versions of Figs. 2 and 4, now featuring the correct data for the UA/SG panel in Fig. 2A and the CDDP/TRITC data panel in Fig. 4A, are shown on the next page. The authors wish to emphasize that the errors made in assembling the data in these figures did not affect the overall conclusions reported in the paper. The authors are grateful to the Editor of *International Journal of Molecular Medicine* for granting them this opportunity to publish a Corrigendum, and apologize to both the Editor and the readership for any inconvenience caused.

## Figures and Tables

**Figure 2:**
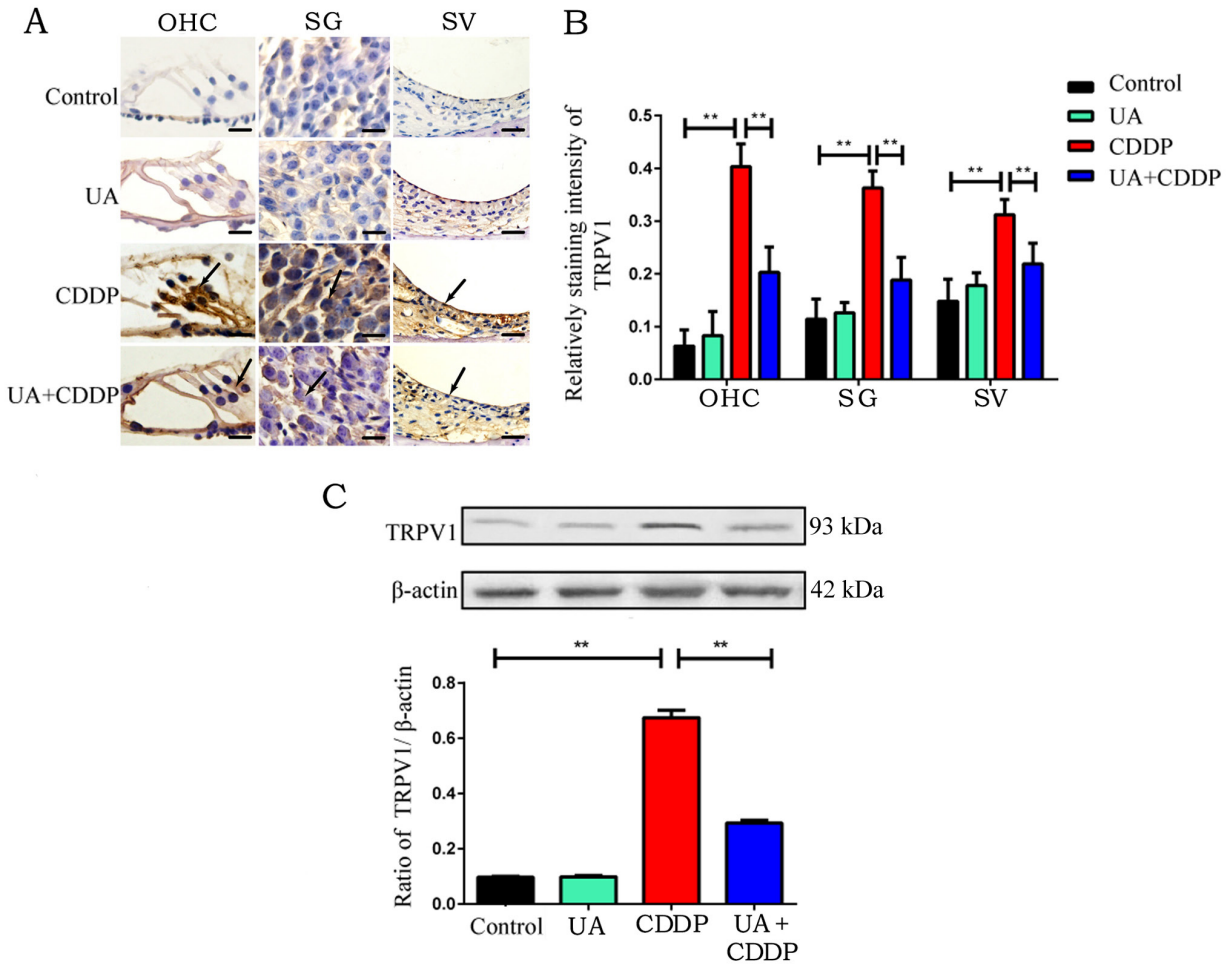
Effect of UA on CDDP-induced expression of TRPV1 in mouse cochlea. (A) Representative immunohistochemical staining images (brown and yellow granules) of TRPV1 on the organ of Corti, SG and SV of the cochleae. Arrows indicate the location of TRPV1-positive expression. Scale bar, 20 *μ*m. (B) Quantification of TRPV1 immunolabeling in the different groups. n=3 ears for each group. ^**^P<0.01. (C) Western blot analysis of TRPV1 was used to detect protein expression of TRPV1 in cochlea, with β-actin as a loading control. n=6 ears for each group. Mean values were obtained from n=3 independent experiments. ^**^P<0.01. UA, ursolic acid; CDDP, cisplatin; SG, spiral ganglion; SV, stria vascularis; TRPV1, transient receptor potential vanilloid receptor 1.

**Figure 4:**
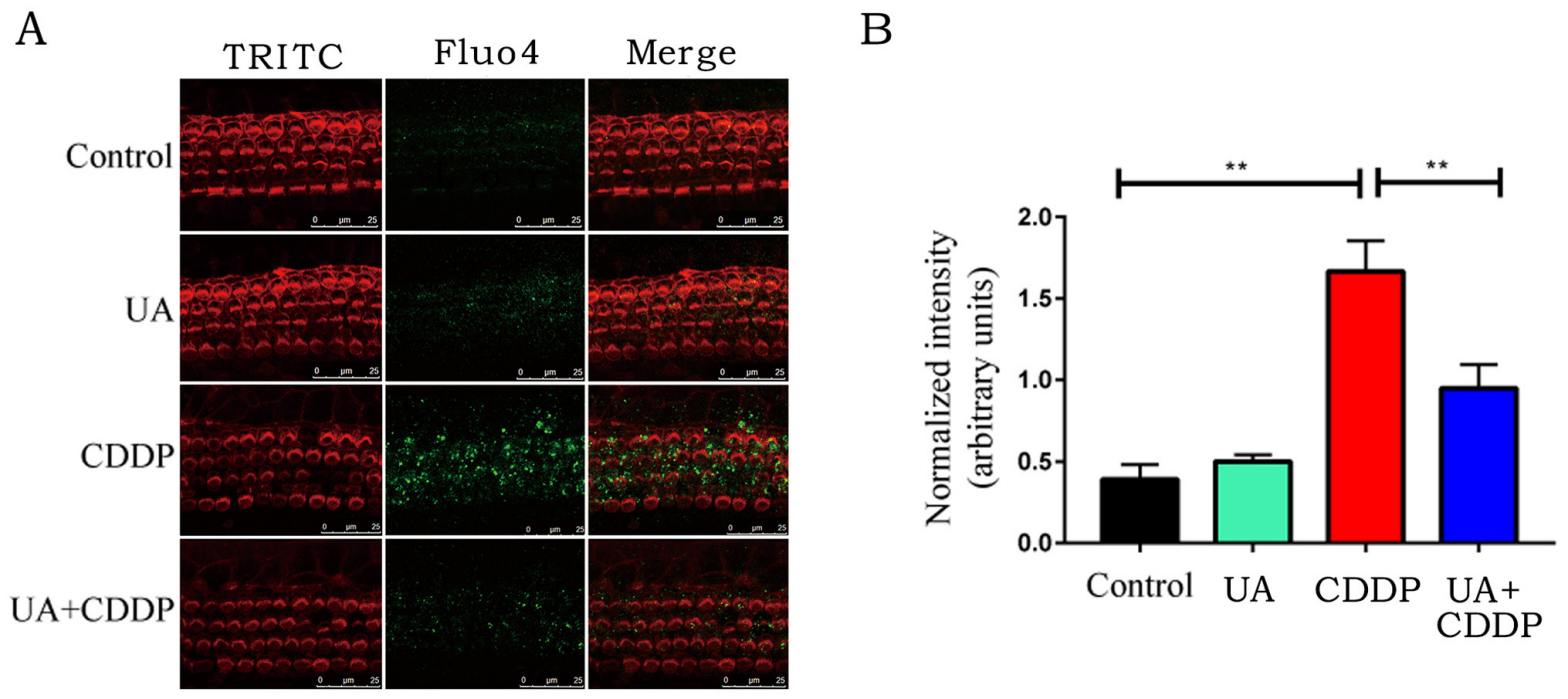
UA inhibits CDDP-induced calcium overload in OHCs. (A) Effect of UA on CDDP-induced measurement of Ca^2+^ levels (green) in OHCs from organotypic culture. OHCs were labeled by TRITC staining (red). Scale bar, 20 *μ*m. (B) Quantitative levels of Fluo-4 Ca^2+^ signals from the cochlea. n=3 ears for each group. Mean values were obtained from n=3 independent experiments. ^**^P<0.01. TRITC, tetramethylrhodamine; UA, ursolic acid; CDDP, cisplatin; OHC, outer hair cell.

